# Inhibitory effects of anthracyclines on partially purified 5′–3′ DNA helicase of *Plasmodium falciparum*

**DOI:** 10.1186/s12936-022-04238-y

**Published:** 2022-07-11

**Authors:** Pongruj Rattaprasert, Pattra Suntornthiticharoen, Paviga Limudomporn, Kanthinich Thima, Porntip Chavalitshewinkoon-Petmitr

**Affiliations:** 1 Department of Protozoology, Faculty of Tropical Medicine, Mahidol University, Ratchawithi Road, Bangkok, 10400 Thailand; 2 Department of Medical Science, Faculty of Science, Rangsit University, Pathumthani, 12000 Thailand; 3 Department of Zoology, Faculty of Science, Kasetsart University, Bangkok, 10900 Thailand

**Keywords:** *P. falciparum* DNA helicase, 5′–3′ *P. falciparum* DNA helicase, DNA helicase inhibitor, Anthracycline

## Abstract

**Background:**

*Plasmodium falciparum* has been becoming resistant to the currently used anti-malarial drugs. Searching for new drug targets is urgently needed for anti-malarial development. DNA helicases separating double-stranded DNA into single-stranded DNA intermediates are essential in nearly all DNA metabolic transactions, thus they may act as a candidate for new drug targets against malarial parasites.

**Methods:**

In this study, a *P. falciparum* 5′ to 3′ DNA helicase (PfDH-B) was partially purified from the crude extract of chloroquine- and pyrimethamine-resistant *P. falciparum* strain K1, by ammonium sulfate precipitation and three chromatographic procedures. DNA helicase activity of partially purified PfDH-B was examined by measuring its ability to unwind ^32^P-labelled partial duplex DNA. The directionality of PfDH-B was determined, and substrate preference was tested by using various substrates. Inhibitory effects of DNA intercalators such as anthracycline antibiotics on PfDH-B unwinding activity and parasite growth were investigated.

**Results:**

The native PfDH-B was partially purified with a specific activity of 4150 units/mg. The PfDH-B could unwind M13-17-mer, M13-31-mer with hanging tail at 3′ or 5′ end and a linear substrate with 3′ end hanging tail but not blunt-ended duplex DNA, and did not need a fork-like substrate. Anthracyclines including aclarubicin, daunorubicin, doxorubicin, and nogalamycin inhibited the unwinding activity of PfDH-B with an IC_50_ value of 4.0, 7.5, 3.6, and 3.1 µM, respectively. Nogalamycin was the most effective inhibitor on PfDH-B unwinding activity and parasite growth (IC_50_ = 0.1 ± 0.002 µM).

**Conclusion:**

Partial purification and characterization of 5′–3′ DNA helicase of *P. falciparum* was successfully performed. The partially purified PfDH-B does not need a fork-like substrate structure found in *P. falciparum* 3′ to 5′ DNA helicase (PfDH-A). Interestingly, nogalamycin was the most potent anthracycline inhibitor for PfDH-B helicase activity and parasite growth in culture. Further studies are needed to search for more potent but less cytotoxic inhibitors targeting *P. falciparum* DNA helicase in the future.

## Background

In 2020, 241 million malaria-infected patients were estimated, of whom 627,000 died, mostly due to *Plasmodium falciparum,* especially in African countries [1]. Although vector control made a significant contribution to the reduction of the global malaria burden, insecticide resistance in *Anopheles* mosquitoes is a recognized threat to malaria control and elimination [1]. Urgent action is needed to slow down the development and further spread of insecticide resistance. Apart from the vector control problem, the lack of complete understanding of the mechanisms of resistance to currently used anti-malarial drugs, the emergence of drug-resistant strains as well as high antigenic variation of parasitic proteins is among the most important reasons for the unsuccessful eradication of this deadly disease. The search for a new approach to the prevention and treatment of the disease is still needed.

Many processes in nucleic acid metabolism, such as replication, repair, recombination, and transcription, need single-stranded (ss) DNA for DNA and RNA polymerases [2]. The family of the enzymes responsible for providing ssDNA during these processes by unwinding duplex is known as DNA helicases. Helicases are ubiquitous and integral members of almost all complexes that catalyze reactions of nucleic acid metabolism [3, 4]. The basic reaction catalyzed by this family of enzymes is the unwinding of the duplex form of DNA or RNA, a processor coupled to the steps of enzymatic nucleoside triphosphate (NTP) hydrolysis. Many DNA and RNA metabolic processes require ssDNA and ssRNA devoid of secondary structures, and these are generated in situ by the action of specific helicases. DNA helicases involved in replication, repair, and recombination work in association with other proteins as part of a complex machine [4]. However, most helicases by themselves can catalyze the strand separation or unwinding process when a suitable DNA or RNA substrate is provided in vitro. The exact mechanism by which helicases accomplish this reaction is not yet clear; however, significant progress has been made. Studies have revealed that helicases are motor proteins that couple NTP hydrolysis to movement on the nucleic acid strand [3, 4].

Approximately 1% of eukaryotic and prokaryotic genome codes for helicases [5]. Helicases can be grouped into distinct classes by their structure and biochemical properties. They can be classified according to their directionality of unwinding, 5′–3′ or 3′–5′, concerning the strand they bind to and move along [6]. Approximately 31 DNA helicases are coded in the human genome [7] while some recombinant *P. falciparum* DNA helicases have been expressed and characterized, including six enzymes in the DEAD-box family, one DEAH enzyme, four RuvB enzymes, UvrD helicase, three RecQ enzymes, XPD helicase, and three parasite-specific helicases (PfPSH1-3) [8–13]. The majority of these recombinant enzymes demonstrate a 3′–5′ direction of unwinding activity concerning the strand of DNA on which the enzyme is bound. Besides the reported recombinant enzymes, conical enzymes should also be investigated during parasite development in infected erythrocytes. *P. falciparum* 3′–5′ DNA helicase (PfDH-A) was successfully purified and characterized from the crude extract of pyrimethamine- and chloroquine-resistant *P. falciparum* (strain K1/Thailand), and known DNA helicase inhibitors, such as anthracyclines, could inhibit PfDH-A helicase activity [14].

Since DNA helicases play important roles in DNA and RNA metabolism, their inhibitors may extend the way to develop new drugs. Various DNA-intercalating compounds have been used to study their effect on *Escherichia coli* DNA helicases [15], simian virus 40 (SV40) large T helicase [16, 17], human DNA helicase II [18], *Plasmodium cynomolgi* DNA helicase [19], some recombinant *P. falciparum* DNA helicases [20–23] and the native *P. falciparum* 3′–5′ DNA helicase [14]. However, *P. falciparum* 5′ to 3′ DNA helicase has not been isolated and purified from parasite crude extract, and the effects of DNA intercalating agents on its unwinding activity have not been examined.

In this study, 5′–3′ DNA helicase of *P. falciparum* (PfDH-B) was partially purified from the crude extract of pyrimethamine- and chloroquine-resistant *P. falciparum* strain and characterized for the substrate preference. The directionality of the partially purified enzyme was also determined, and its sensitivity to known DNA helicase inhibitors, including anthracyclines, was investigated.

## Methods

### Parasite culture

*Plasmodium falciparum* strain K1, chloroquine- and pyrimethamine- resistant strain from Thailand [24], was cultivated using a large-scale culture method [25]. After synchronization by 5% sorbitol treatment [26], the cultivation of *P. falciparum* was started with 2% parasitaemia of ring form and 2% haematocrit of human red blood cells (group O) in complete medium (RPMI 1640, 5.94 g HEPES, 5% NaHCO_3,_ and 10% human serum) at 37 °C in 5% CO_2_ incubator. *Plasmodium falciparum* cultures containing mostly trophozoite and schizont stages were harvested when parasitaemia reached more than 20%. The harvested erythrocytes were lysed by 0.15% (w/v) saponin in phosphate buffer saline (PBS) pH 7.6 at 37 °C for 20 min. The suspension was washed with PBS by centrifugation at 664 ×*g*, 25 °C for10 min. The parasite pellet was obtained and kept at − 80 °C until use. Approximately one ml of parasite pellet was obtained from 1 L of the culture, therefore, 5 L of culture was performed to obtain 5 ml of packed parasite pellet for enzyme purification.

### Partial purification of *Plasmodium falciparum* 5′ to 3′ DNA helicase (PfDH-B)

All the purification steps were conducted on ice or at + 4 °C. Approximately 10^11^ parasites (5 ml packed cells) were suspended in 5 volumes of extraction buffer (50 mM Tris HCl (pH 7.6), 1 mM ethylenediaminetetraacetic acid (EDTA), 2 mM dithiothreitol (DTT), 0.01% Nonidet P40 (NP_40_), 1 mM phenylmethylsulfonyl fluoride (PMSF)), ground in a Dounce homogenizer and diluted with an equal volume of dilution buffer (50 mM Tris HCl (pH 7.6), 1 mM EDTA, 2 mM DTT, 20% (w/v) sucrose, 0.01% NP_40_, 1 mM PMSF). To extract nucleoproteins, 3 M KCl was added with stirring until a final concentration of 0.5 M KCl was obtained. After stirring for 30 min, the suspension was centrifuged at 100,000 × *g* for 40 min. The supernatant was dialyzed overnight against buffer A (25 mM Tris HCl (pH 8.0), 1 mM EDTA, 1 mM PMSF, 1 mM DTT, 5% sucrose, 20% glycerol, 0.01% NP_40_) and designated Fraction A. Fraction A was loaded onto a 6 ml Resource Q column equilibrated with buffer A. The column was washed with 54 ml of buffer A, and proteins were eluted with a 60 ml linear gradient (0–1 M KCl in buffer A) at a flow rate of 2 ml/min, and 2 ml fractions were collected. The active fractions, 11–23, were pooled and designated as Fraction B. Fraction B was dialyzed overnight against buffer B (buffer A adjusted to pH 7.5) and loaded onto a 1 ml Mono S column. The column was washed with 10 ml of buffer B, and the enzyme was eluted with an 8 ml linear gradient (0–1 M KCl in buffer B) at a flow rate of 0.25 ml/min, and 0.25 ml fractions were collected. The active fractions, 11–13, from the Mono S column were pooled and designated as Fraction C. Fraction C was dialyzed against buffer B and applied to a 1 ml ssDNA column. The column was washed with 10 ml of buffer, and protein was eluted with a 5 ml linear gradient (0–1 M KCl in buffer B) at a flow rate of 0.25 ml/min, and 0.15 ml fractions were collected. The active fractions, 14–17, containing 5′ to 3′ DNA helicase, was eluted and designated as Fraction D. The protein concentration in each fraction was measured by Bradford’s assay (Bio-Rad Laboratories, Hercules, California).

### Substrate preparation for standard DNA helicase assay

All oligodeoxynucleotides were synthesized by BioService Unit, National Science and Technology Development Agency, Bangkok, Thailand. The standard substrate used in DNA helicase assay consisted of ^32^P-labelled complementary oligodeoxynucleotide 1 (Oligo-1, 5′-GTAAAACGACGGCCAGT-3′) annealed to M13mp19 ( +) strand (Invitrogen, Waltham, Massachusetts) to create a M13-17-mer partial duplex [27]. The above Oligo-1 (100 ng) was labelled at 5′-end with T4 polynucleotide kinase and 250 mCi [γ-^32^P]ATP (PerkinElmer, Waltham, Massachusetts). The ^32^P-labelled oligodeoxynucleotide was annealed with 2.5 mg of M13mp19 (+) strand in 20 mM Tris–HCl (pH 7.5), 10 mM MgCl_2_, 100 mM NaCl, and 1 mM DTT by heating at 95 °C for 1 min, transferred immediately to 65 °C for 2 min, and then cooled slowly to 25 °C for 30 min. Non-hybridized oligonucleotides were removed by passage through Microspin S-400 column (GE Healthcare, Amersham, UK), followed by an Autoseq 50 column (GE Healthcare, Amersham, UK).

### DNA helicase assay

The standard DNA helicase assay measures the unwinding of α ^32^P-labelled DNA fragments from a partial M13-17-mer duplex. The reaction mixture (10 ml) containing 20 mM Tris–HCl (pH 9.0), 8 mM DTT, 2 mM MgCl_2_, 2 mM ATP, 10 mM KCl, 4% (w/v) sucrose, 80 mg/ml bovine serum albumin (BSA), ^32^P-labelled helicase substrate (1000 counts per minute [cpm]) and 2 ml of parasite protein from each fraction was incubated at 37 °C for 20 min. The reaction was terminated with 10 ml of loading dye (10% ficoll, 50 mM EDTA, 10 mM Tris–HCl, 0.25% xylene cyanol, 0.25% bromophenol blue). The substrate and product were separated by electrophoresis in a 20% non-denaturing polyacrylamide gel containing 0.5 × Tris HCl-boric acid-EDTA (TBE) (5.4 g Tris base, 2.75 g boric acid, and 0.465 g EDTA in 1 L of deionized water) at 92 V for 1 h and 40 min (Mini-Protean II Dual Slab Cell, Bio-Rad). The gel was exposed to X-ray film to identify the location of the radiolabelled product, which was then excised from the gel. The incorporated radioactivity was measured using a MicroBeta TriLux Liquid Scintillation Counter (PerkinElmer, Waltham, Massachusetts).

One unit of DNA helicase activity is defined as the amount of enzyme that unwinds 1% of the DNA helicase substrate in 20 min at 37 °C.

### Directionality of *P. falciparum* DNA helicase

Oligodeoxynucleotide 2 (Oligo-2, 50 ng 5′-TCGAGCTCGGTACCCGGGGATCCTCTAGAGTCGACCTGCAGG-3′) was labelled at the 5′ end with [γ^32^P]ATP and annealed with M13mp19 ( +) strand in 20 mM Tris–HCl (pH 7.5), 10 mM MgCl_2_, 100 mM NaCl and 1 mM DTT. The partial duplex was passed through a 1 ml Microspin S-400 and once through an Autoseq G50 column to remove unincorporated radionucleotides. The annealed DNA was then labelled at 3' end by incubating with 10 mCi [α^32^P]dCTP and 5 units of DNA polymerase I (Invitrogen, Waltham, Massachusetts) at 23 °C for 20 min. Then, 50 mM unlabelled dCTP was added, and the solution was further incubated at 23 °C for 20 min. The unincorporated radionucleotides were removed by passing through a 1 ml Microspin S-400 column. The annealed duplex was digested with *Sma*I (Invitrogen, Waltham, Massachusetts) at 25 °C for 1 h. Unwinding in the 5′ to 3′ direction was detected by monitoring the release of the radioactive 28-mer while unwinding in the 3′ to 5′ direction by the release of the radioactive 15-mer from the duplex substrate in 20% nondenaturing polyacrylamide gel.

### Substrate preference of *P. falciparum* 5′ to 3′ DNA helicase

#### Preparation of blunt-ended duplex substrate

Oligodeoxynucleotide 3 (Oligo-3, 50 ng of 41-mer; 5′-AATTCGAGCTCGGTACCCGGGGATCCTCTAGAGTCGACCTG-3′) was labelled at the 5′ end with T4 polynucleotide kinase and [γ^32^P]ATP (250 mCi). The labelled fragment was annealed with oligodeoxynucleotide 4 (Oligo-4, 50 ng 5′-CAGGTCGACTCTAGAGGATCCCCGGGTACCGAGCTCGAATT-3′) in 40 mM Tris–HCl (pH 7.5), 20 mM MgCl_2_, and 50 mM NaCl at 65 °C for 2 min and allowed to stand at 25 °C for 30 min. The solution was passed through a 1 ml Microspin S-400 column to remove unincorporated oligonucleotides.

#### Preparation of M13-31-mer with hanging tail at 3′ or 5′ end

Oligodeoxynucleotide 5 (Oligo-5, 50 ng 5′-GTTCCAGCGCTAGCTTCG AGCTCGGTACCCGGGGATCCTCTAGAG-3′) was used to prepare M13-31-mer with hanging tail at 5′ end. Oligonucleotide 6 (Oligo-6, 50 ng 5′-TCGAGCTCGGTACCCGGGGATCCTCTAGAG(T)_14_–3′ was used to prepare M13-31-mer with hanging tail at 3′ end. Oligo-5 and Oligo-6 were labelled at 5′-end with T4 polynucleotide kinase and [γ-^32^P]ATP (250 mCi) and then annealed with 1.25 mg of single-stranded circular M13 mp19 ( +) strand DNA in 20 mM Tris–HCl (pH 7.5), 10 mM MgCl_2_, 100 mM NaCl and 1 mM DTT by heating at 95 °C for 1 min, transferring immediately to 65 °C for 2 min, and then cooling slowly to 25 °C for 30 min. Non-hybridized oligodeoxynucleotides were removed by gel filtration through a 1 ml of Microspin S-400 column.

#### Preparation of linear substrate with 3′ end hanging tail

Oligodeoxynucleotides 7 (Oligo-7, 50 ng 5′-(T)_10_CGAGCTCGGTACCCGGGGATCCTCTAGAGTCGACCTGCA(T)_11_–3′) was prepared for a linear substrate with 3′ and 5′ end hanging tails. Fifty nanograms of Oligo-5 were labelled at 5′-end, annealed with 1.25 mg of M13mp19 (+) strand DNA, and then digested with *SmaI* to generate a 24-mer product. Fifty nanograms of Oligo-5 were annealed with 1.25 ng of M13mp19 (+) strand, labelled at the 3′ end, and then digested with *SmaI* to generate a 36-mer product.

#### Inhibition of *P. falciparum* 5′ to 3′ DNA helicase

Aclarubicin, daunorubicin, doxorubicin, and nogalamycin were purchased from Sigma-Aldrich, St. Louis, Missouri. Stock solutions (10 mM) of the drugs were prepared in dimethylsulfoxide (DMSO) and stored at − 20 °C until use. Drugs were diluted with 10 mM Tris–HCl, pH 9. M13-17-mer DNA substrate (1000 cpm) was incubated with appropriate concentrations of drugs for 10 min before proceeding to the helicase assay, which contained 2 ml (0.2 mg) of parasite enzyme. The highest concentration of 0.2% (v/v, 28 mM) DMSO was used as a negative control.

#### In vitro sensitivity test of *P. falciparum* to inhibitors

In vitro drug assays were performed in 96-well tissue culture plates. In this experiment, *P. falciparum* strain K1 was used and the initial parasitaemia was 1% of the ring form after synchronization by 5% sorbitol treatment [26]. Twenty-five microlitres of the drug at concentrations of 0.09–0.9 mM were added to 200 ml of 1.5%, erythrocyte suspension in a complete RPMI medium to obtain the final drug concentrations of 0.01–0.1 mM. The highest concentration of 0.2% (v/v, 28 mM) DMSO was tested as a control. Cultures were incubated at 37 °C for 24 h in a CO_2_ incubator, and then 0.25 mCi of (^3^H) hypoxanthine (6.2 Ci/mmol, ThermoFisher, Waltham, Massachusetts) was added to each well, and 24 h further incubation was done [28]. Erythrocytes were harvested by using an automated sample harvester, and the radioactivity incorporated into the parasites was measured. A single experiment was performed for each anthracycline, and individual drug concentration was tested in triplicate. IC_50_ was defined as 50% inhibition of the incorporation of (^3^H) hypoxanthine into parasites compared with the control. The dose–response curves of (^3^H) hypoxanthine incorporation and drug concentrations were generated, and IC_50_ was obtained using SigmaPlot 12.0.

## Results

### Partial purification of *P. falciparum* 5′ to 3′ DNA helicase

**Fig. 1:**
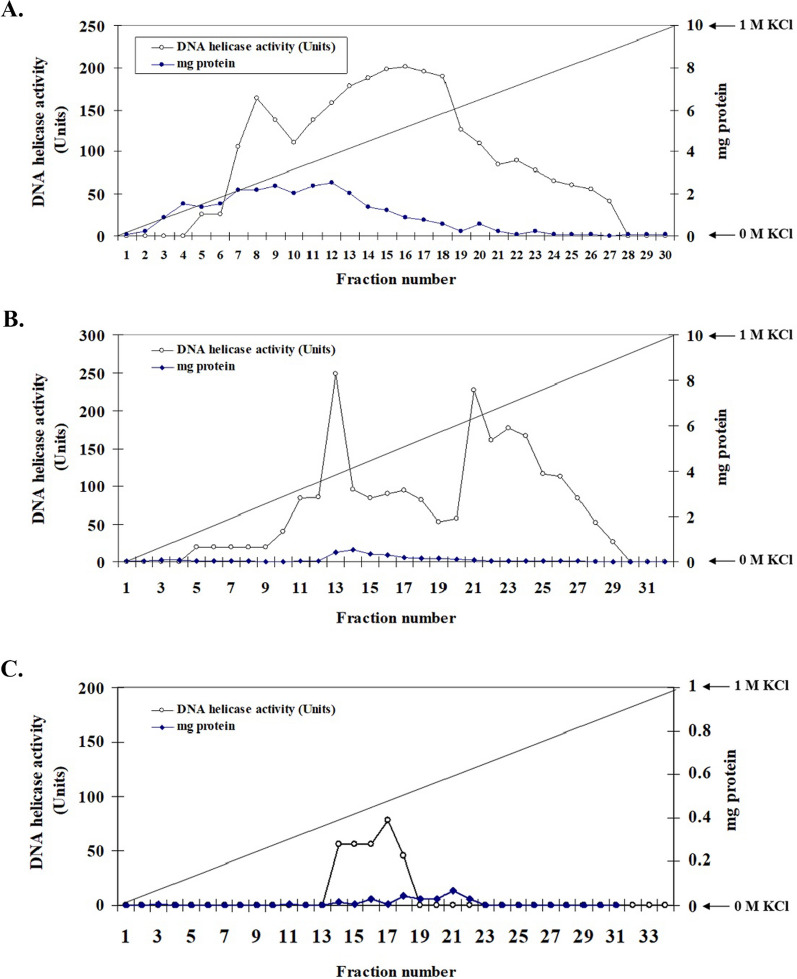
Partial purification of native 5′ to 3′ DNA helicase from *P. falciparum* crude extract. **A** Parasite crude extract was purified on the Resource Q column with 0–100% KCl linear gradient. **B** Resource Q pooled fraction containing helicase activity was purified on the Mono S column with 0–100% KCl linear gradient. **C** Mono S pooled active fraction was further purified on the ssDNA column with 0–100% KCl linear gradient. Each fraction was measured for the amount of proteins by using the Bradford reagent and assayed for unwinding activity using the M13-17-mer substrate

*Plasmodium falciparum* 5′ to 3′ DNA helicase was partially purified from the crude extract using fast protein liquid chromatography (FPLC). The crude extract (Fraction A) was loaded onto the Resource Q column (6 ml), and thirty fractions were collected and assayed for DNA helicase activity. The active fractions (fraction number 11–23) were eluted at 0.23–0.6 M KCl in buffer A and pooled (Fraction B) (Fig. 1A). Fraction B was dialyzed in buffer B and applied onto the Mono S column (1 ml) equilibrated with buffer B. A total of 32 fractions were collected and assayed for DNA helicase activity. The active fractions (fraction number 11–13) were eluted at 0.12–0.23 M NaCl in buffer B and pooled (Fraction C) (Fig. 1B). Fraction C was then dialyzed in buffer B and loaded onto the ssDNA column (1 ml) equilibrated with buffer B. A total of 34 fractions were collected and assayed for DNA helicase activity. The active fractions (fraction number 14–17) were eluted at 0.09–0.2 M KCl in buffer B and pooled (Fraction D) (Fig. 1C).

**Fig. 2:**
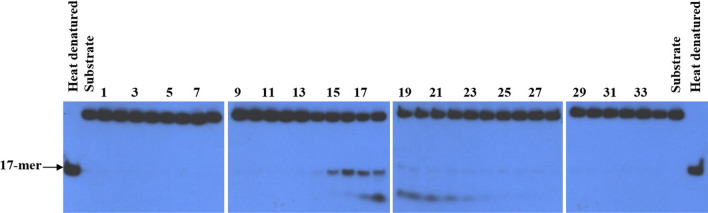
Unwinding activity of partially purified *P. falciparum* 3′ to 5′ DNA helicase eluted from ssDNA column (Fraction D). The standard displacement assay with M13-17-mer substrate and 2 ml of the enzyme from each fraction was done. Numbers above the figure are the fraction eluted from the column

The M13-17 mer substrate was used to determine the 5′ to 3′ unwinding activity of *P. falciparum* 5′ to 3′ DNA helicase during the purification steps. The amount of 5′ to 3′ DNA helicase, specific activity, and yield after each purification step are shown in Table 1. From 5 ml of parasite pellet, only 0.06 mg of 5′ to 3′ DNA helicase enzyme was obtained with a specific activity of 4,150 units/mg. DNA helicase was co-purified with nuclease from the Resource Q column. Mono S column was an important step to separate 5′ to 3′ DNA helicase from 3′ to 5′ DNA helicase of *P. falciparum*, and ssDNA column was able in turn to separate 5′ to 3′ DNA helicase from contaminating nuclease activity (Fig. 2).

**Table 1:** Partial purification of *P. falciparum* 5′ to 3′ DNA helicase from crude extract

Fractions	Total protein (mg)	Total activity (units)	Specific activity (units/mg)
Crude extract	123.52	ND	ND
Resource Q (f 11–23)	13.14	1940	147
Mono S (f 11–13)	0.52	437	840
ssDNA (f 12–16)	0.06	246	4,150

**Fig. 3:**
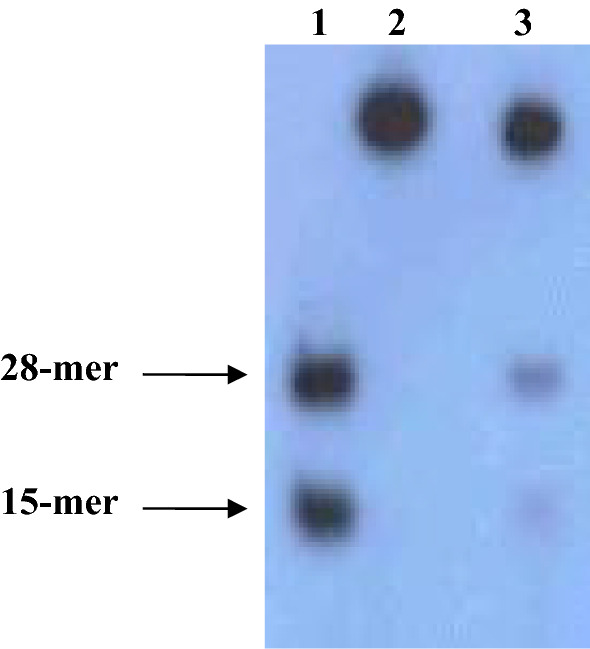
Directionality of *P. falciparum* 5′ to 3′ DNA helicase activity. The 43-mer linear partial duplex was used as a substrate and then digested with *Sma*I. Lane 1 corresponds to a heat-denatured substrate, lane 2 is negative control (reaction without enzyme), and lane 3 is a reaction with 0.2 mg of partially purified *P. falciparum* 5′–3′ DNA helicase

PfDH-B demonstrated 5′ to 3′ directionality as the 28-mer product was clearly seen (Fig. 3). Since it is a partially purified enzyme, a residual amount (< 5%) of the 3′–5′ DNA helicase enzyme could be observed with the faint band of the 15-mer product on an autoradiogram (Fig. 3).

### Determination of substrate preference

**Fig. 4:**
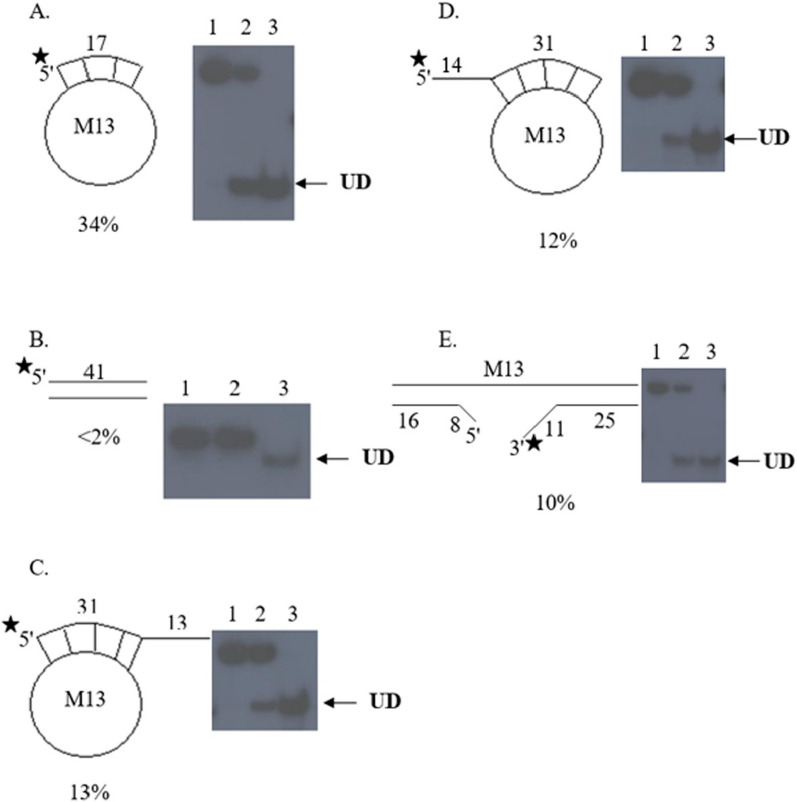
Unwinding activity of *P. falciparum* 5′ to 3′ DNA helicase with different substrates. **A** the M13-17-mer partial duplex. **B** the 41-mer blunt end. **C** the M13-44-mer with 3′ hanging tail. **D** the M13-45-mer with 5' hanging tail. **E** the 5′ to 3′ hanging tails on the same M13 template. Asterisks denote the ^32^P-labelled end. Lane 1 is the control without enzyme, Lane 2 is a reaction with partially purified enzyme, and Lane 3 is the heat-denatured substrate. UD: unwound DNA

The substrate preferences of *P. falciparum* 5′ to 3′ DNA helicase are shown in Fig. 4. Under the experimental conditions, *P. falciparum* 5′ to 3′ DNA helicase could completely unwind 34% of the M13-17-mer (Fig. 4A). A 41-mer blunt end substrate was not a substrate for unwinding activity (Fig. 4B). When the annealed length to the M13 template was increased to 31 bp, unwinding activity was reduced by more than 65% (compare Fig. 4C and D with Fig. 4A), and unwinding activity was not influenced by the types of hanging tail. To confirm the 5′ to 3′ unwinding direction of *P. falciparum* 5′ to 3′ DNA helicase, the substrate containing both 5′ to 3′ hanging tails on the same M13 template (Fig. 4E) was used in the unwinding assay, and only the 36-mer strand was displaced (10%) and not the 24-mer strand.

### Inhibition of *P. falciparum* 5′ to 3′ DNA helicase activity and parasite growth with anthracyclines

**Fig. 5:**
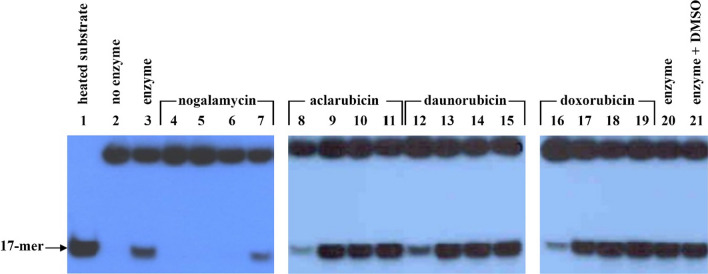
Inhibitory effects of anthracyclines on *P. falciparum* 5′–3′ DNA helicase. Lane 1 is the heat-denatured substrate. Lane 2 is negative control and lanes 3 and 20 are positive controls. Lanes 4–7 are nogalamycin at concentrations of 10, 5, 1, 0.1 μM, respectively. Lanes 8–19 are aclarubicin, daunorubicin, and doxorubicin at the same series of drug concentrations at 10, 1, 0.1, 0.01 μM, respectively. Lane 21 is an enzyme with 0.2% (v/v) DMSO

Anthracycline antibiotics were tested for their inhibitory effects on *P. falciparum* 5′ to 3′ DNA helicase under the standard DNA unwinding assay. The IC_50_ values of aclarubicin, daunorubicin, doxorubicin, and nogalamycin were 4.0, 7.5, 3.6, and 3.1 μM, respectively (Table 2 and Fig. 5). Moreover, all compounds exhibited inhibitory effects on parasite growth with different IC_50_s as shown in Table 2. The most potent inhibitor was nogalamycin showing the mean (± standard deviation) IC_50_ of 0.1 ± 0.002 μM, whereas the highest IC_50_ was found in daunorubicin with IC_50_ of 2.5 ± 0.16 μM. Aclarubicin and doxorubicin showed IC_50s_ of 1.8 ± 0.75 and 1.5 ± 0.21 μM, respectively, which were comparable to chloroquine (IC_50_ = 1.7 ± 1.68 μM) (Table 2).

**Table 2:** Effects of anthracycline antibiotics on unwinding activity of PfDH-B and in vitro parasite growth

Drugs	IC_50_ (μM)	
	PfDH-B	*P. falciparum* strain K1*
Aclarubicin	4.0	1.8 ± 0.75
Daunorubicin	7.5	2.5 ± 0.16
Doxorubicin	3.6	1.5 ± 0.21
Nogalamycin	3.1	0.1 ± 0.002
Chloroquine	ND	1.7 ± 1.68

*ND* not done

^*^The apparent IC_50_ value was determined from the concentration of the inhibitor sufficient to achieve 50% growth inhibition. The values are the mean (± standard deviation) obtained from a single assay of each drug in triplicate

## Discussion

All living organisms contain a variety of DNA helicases due to the diversity of DNA transactions that require at least transiently, a ssDNA structure. For instance, 9 DNA helicases (HDH I-IX) have been purified from HeLa cells [29]. In *P. falciparum*, at least 19 recombinant DNA helicases were studied for their biochemical and functional properties [8–13]. It is also worth noting that there is only one report of *P. falciparum* 3'-5' DNA helicase (PfDH-A) purified from parasite crude extract [14].

In this study, *P. falciparum* 5′ to 3′ DNA helicase was partially purified from parasite crude extract using a series of columns in FPLC. The partially purified 5′ to 3′ DNA helicase contained a residual of 3′ to 5′ helicase activity which was possibly due to contaminating 3′ to 5′ helicase of the parasite enzyme. Due to low recovery of *P. falciparum* 5′ to 3′ DNA helicase (0.05% yield from 5 ml of packed parasites), characterization of the enzyme was limited to its substrate preferences and inhibition by a limited number of anticancer anthracyclines. Alternatively, the expression of corresponded genes in prokaryotic and eukaryotic expression systems can probably produce amounts of purified enzymes that can never be purified directly from parasites. However, the recombinant enzymes were expressed from 19 out of 31 genes encoded by *P. falciparum* DNA helicases so far, and only three recombinant proteins (PfRuvB1, PfRuvB2, and PfXPD) showed 5′ to 3′ directionality [30–32]. Since these recombinant enzymes did not cover all 5′ to 3′ *P. falciparum* helicases that exist during parasite development, therefore, the purification of the enzyme from parasite crude extract was the suitable approach for studying the sensitivity of 5′ to 3′ *P. falciparum* helicase to anthracyclines in this study.

*Plasmodium falciparum* 5′ to 3′ DNA helicase could unwind a short 17-mer oligonucleotide annealed to circular ssDNA (M13-17-mer), but its unwinding activity was reduced by more than half when a 31-mer with hanging 3' or 5' tail was used. Unwinding activity of *P. falciparum* 5′ to 3′ DNA helicase is about 10% when linear substrate with 3' end hanging tail was used (Fig. 4E), indicating that this enzyme does not need a fork-like structure substrate. *Plasmodium falciparum* 5′ to 3′ DNA helicase shared some characteristics with human DNA helicase (HDH IV). The similar characteristics were the direction of movement of 5′ to 3′ polarity and no requirement of a replication fork-like structure to perform the unwinding. However, *P. falciparum* 5′ to 3′ DNA helicase unwound slightly an M13-31-mer with hanging tail at 3' or 5' end whereas HDH IV cannot unwind a 25-mer or longer duplex [33].

*Plasmodium falciparum* 5′ to 3′ DNA helicase did not unwind blunt-ended substrates as also found in PfRecQ1 [12], PfRuvB3 [13], indicating a requirement of the enzyme for an initial binding site composed of ssDNA like most DNA helicases, including *E. coli* DNA helicases [34, 35]. However, *E. coli* DNA helicase II [35] and large T antigen of SV40 [36] can unwind fully duplex substrates.

PfDH-B unwinds DNA in the 5′ to 3′ direction, which is similar to PfRuvB1, PfRuvB2, and PfXPD [30–32]. The results reveal that PfRuvB1, PfRuvB2, and PfRuvB3 bind to ssDNA in vitro [31]. Moreover, PfRuvB1 possesses ssDNA-stimulated ATPase activity [30] while PfXPD showed ssDNA-dependent ATPase and helicase activities [32]. PfDHB may be involved in several cellular processes, such as transcription and cell cycle progression, as found in RuvB family members and the nucleotide excision repair as PfXPD [8]. For DNA helicase involved in DNA replication, the polarity of the reaction is strongly indicative of the location of the helicase on the leading (3′ to 5′ polarity) or lagging (5′ to 3′ polarity) strand. The large T7 of SV40 shows a 5′ to 3′ polarity, moving along with the replisome-primosome complex, unwinding DNA in front of the incoming replicating DNA polymerase on the leading strand, and preparing a suitable substrate for primase on the lagging strand [37]. In the herpes simplex virus, a helicase-primase complex appears to move along the growing fork unwinding in the 5′ to 3′ direction in a way similar to what has been observed in prokaryotes [38]. *Plasmodium falciparum* primase has been identified [39], but it remains to be shown whether the 5′ to 3′ DNA helicase obtained in this study also works together with the primase as the DNA replicating fork.

DNA helicases have been targets for chemotherapy. The DNA-intercalating anthracycline drugs stabilize duplex DNA and increase the energy required to separate paired DNA strands, thereby inhibiting DNA helicase unwinding ability [40]. Aclarubicin, daunorubicin, doxorubicin, and nogalamycin inhibit *P. falciparum* 5′ to 3′ DNA helicase activity with IC_50_ values ranging from 3.1 to 7.5 μM (Table 2). The most potent compound in the present study, nogalamycin, had an IC_50_ value of 3.1 μM, which is lower than that reported for bipolar helicases, PfUDN and PfD66/PfDDX19 (IC_50_s = 3.5 and 5 μM, respectively) [22], but higher than that seen with recombinant 3′–5′ helicase, PfH45 (IC_50_ = 0.5 μM) [20] and human DNA helicase HDH II (IC_50_ = 0.42 μM) [14]. Our results indicate that anthracyclines inhibited both 3′–5′ and 5′–3′ DNA helicases of *P. falciparum* and that PfDH-B was more sensitive to nogalamycin than PfDH-A (IC_50_ = 5 μM) [14]. It is worth noting that the M13-17-mer substrate contains several GC sequences that are the preferred binding sites for daunorubicin and nogalamycin [41]. Moreover, all tested anthracyclines inhibited the growth of *P. falciparum* K1 strain, with IC_50_ values ranging from 0.1 to 2.5 µM (Table 2). Since the cytotoxicity test of these compounds was not conducted in the present study, anthracyclines should be tested compared with their human counterparts to explore the possibility to develop less cytotoxic drug candidates in the future.

In comparison to PfDH-A, besides the difference in the directionality, PfDH-B differs from PfDH-A in substrate preference because PfDH-B does not prefer a fork-like substrate as found in PfDH-A. Furthermore, nogalamycin was the most potent inhibitor of the unwinding activity of PfDH-B whereas daunorubicin was the most active compound for PfDH-A when tested by the same set of anthracyclines [14]. However, both enzymes demonstrated the similarity in the inability to use a blunt-end duplex as a substrate.

The anthracycline antibiotics have several well-described biological actions, such as the inhibition of enzymes (DNA polymerases [42, 43], RNA polymerases [44], topoisomerase II [45], DNA ligase [46], and DNA repair enzymes [47]), production of free radicals [48], modulation of membranes [49], anticancer activities [50–52]. Besides the above biological actions, certain anthracyclines have been shown to inhibit helicase in vitro*,* notably the minor groove binding bis-benzimidazoles, and also the intercalating anthracyclines [13, 53]. The isolation and purification of *P. falciparum* 5′ to 3′ DNA helicase may open up the possibility of screening for effective and specific compounds targeting the *P. falciparum* DNA helicase, which might help expand the existing limited armory of anti-malarials.

## Conclusions

The large-scale culture of *P. falciparum* permitted the partial purification and characterization of native PfDH-B. The differences between PfDH-B and PfDH-A in substrate preference and sensitivity to known DNA helicase inhibitors, such as nogalamycin and daunorubicin, were observed. Based on this preliminary result, further studies should be performed to investigate more effective compounds with the least toxic *P. falciparum* DNA helicase inhibitors in the future.

## Data Availability

Data supporting results in the article are available from the corresponding author upon request.

## Abbreviations

cpm: Count per minute
Ci: Curie
µCi: Microcurie
µg/ml: Micrograms per milliliter
µl: Microlitre
L: Liter
µM: Micromolar
^32^P: Phosphorus-32
BSA: Bovine Serum Albumin
dCTP: Deoxycytidine triphosphate
DMSO: Dimethylsulfoxide
DNA: Deoxyribonucleic acid
DTT: Dithiothreitol
EDTA: Ethylenediaminetetraacetic acid
FPLC: Fast protein liquid chromatography
HDH IV: Human DNA helicase IV
HEPES: (4-(2-Hydroxyethyl)-1-piperazineethanesulfonic acid)
h: Hour
IC_50_: The half-maximal inhibitory concentration
M: Molar
mg: Milligram
min: Minute
ml: Milliliter
ml/min: Milliliters per minute
mM: Millimolar
ng: Nanogram
NP_40_: Nonidet P40
NTP: Nucleoside triphosphate
PfDH-A: *P. falciparum* 3′–5′ DNA helicase
PfDH-B: *P. falciparum* 5′–3′ DNA helicase
PMSF: Phenyl methyl sulfonyl fluoride
RNA: Ribonucleic acid
ssDNA: Single-stranded deoxyribonucleic acid
SV40: Simian virus 40
TBE: Tris HCl-boric acid-EDTA
w/v: Weight by volume
x*g*: Relative centrifugal force
